# The potential contribution of miRNA-200-3p to the fatty acid metabolism by regulating *AjEHHADH* during aestivation in sea cucumber

**DOI:** 10.7717/peerj.5703

**Published:** 2018-10-02

**Authors:** Muyan Chen, Shanshan Wang, Xingke Li, Kenneth B. Storey, Xiumei Zhang

**Affiliations:** 1 Key Laboratory of Mariculture (Ocean University of China), Ministry of Education, Ocean University of China, Qingdao, China; 2 Institute of Biochemistry, Carleton University, Ottawa, Canada

**Keywords:** Sea cucumber, Fatty acid metabolism, *AjEHHADH*, Aestivation

## Abstract

The sea cucumber (*Apostichopus japonicus*) has become a good model organism for studying environmentally-induced aestivation by a marine invertebrate more recently. In the present study, we hypothesized that miRNA-200-3p may contribute to establish rapid biological control to regulate fatty acid metabolism during a estivation. The peroxisomal bi-functional enzyme (EHHADH) is a crucial participant of the classical peroxisomal fatty acid *β*-oxidation pathway, the relative mRNA transcripts and protein expressions of EHHADH were analyzed in intestine from sea cucumbers experienced long-term aestivation. Both mRNA transcripts and protein expressions of EHHADH in intestine decreased significantly during deep-aestivation as compared with non-aestivation controls. Analysis of the 3′ UTR of *AjEHHADH* showed the presence of a conserved binding site for miR-200-3p. Level of miR-200-3p showed an inverse correlation with EHHADH mRNA transcripts and protein levels in intestine, implicating miR-200-3p may directly targeted *AjEHHADH* by inducing the degradation of *AjEHHADH* mRNA in the aestivating sea cucumber, further dual-luciferase reporter assay validated the predicted role of miRNA-200-3p in regulating *AjEHHADH*. In order to further understand their regulatory mechanism, we conducted the functional experiment in vivo. The overexpression of miR-200-3p in sea cucumber significantly decreased mRNA and protein expression levels of *AjEHHADH*. Taken together, these findings suggested the potential contribution of miRNA-200-3p to the fatty acid metabolism by regulating *AjEHHADH* during aestivation in sea cucumber.

## Introduction

The sea cucumber, *Apostichopus japonicus*, is an important species farmed in the Chinese aquaculture industry and recently has been embraced as a model organism for studying aestivation in marine invertebrates (Chen et al., 2013; Chen & Storey, 2014; Chen, Zhu & Storey, 2016; Chen et al., 2016). This animal becomes inactive when water temperature rises over 18 °C and is induced to aestivation when temperatures rise over 25 °C during the summer; thereafter, the animals remain in dormancy for over 100 days without feeding or locomotion (Sui et al., 1985; Sui & Liao, 1988; Yang et al., 2005). During prolonged periods of inactivity, *A. japonicus* undergoes a series of physiological and biochemical adaptations characterized by profound metabolic rate depression, loss of body weight, intestinal atrophy, and global redistribution of metabolic fuels between tissues (Yang et al., 2006; Yuan et al., 2007). Upon recovery from aestivation, all of these features, including intestinal atrophy, are reversed. Numerous studies completed by our group have outlined the molecular mechanisms of natural aestivation tolerance in sea cucumbers. These studies investigated the roles and regulation involved in providing global suppression of transcription and translation in order to minimize energy expenditure and protect cellular functions (Chen, Zhu & Storey, 2016; Chen et al., 2016; Chen et al., 2013; Chen & Storey, 2014). The optimal regulatory mechanisms for aestivation survival are those that can broadly control multiple metabolic processes, can be coordinated by extracellular stimuli, are easily induced and readily reversed. Among these mechanisms, post-transcription regulation of various transcripts by microRNAs (miRNAs) has become a topic of interest for many researchers.

MiRNAs are highly conserved, small non-coding RNA molecules that can regulate gene expression by binding to the 3′-untranslated regions (UTRs) of target genes, resulting in translational suppression by either causing the degradation of the corresponding mRNA transcript or move it into storage in p-bodies or stress granules for future use (Bartel, 2004; Biggar & Storey, 2011). Recently, miRNA studies have outlined the role of microRNAs in supporting hypometabolic states such as hibernation in ground squirrels and bats (Morin, Dubuc & Storey, 2008; Kornfeld, Biggar & Storey, 2012; Maistrovski, Biggar & Storey, 2012), freeze tolerance by wood frogs (Biggar, Dubuc & Storey, 2009), anoxia tolerance in turtles and marine snails (Biggar & Storey, 2012; Biggar et al., 2012) and aestivation in sea cucumbers (Chen et al., 2013; Chen & Storey, 2014). In periods where energy availability is limited, it is vitally important to rapidly and readily change the energy expenditure of the cell to provide sufficient supply for pro-survival processes while suppressing harmful or unnecessary processes in a reversible and coordinated fashion. This regulation can be achieved by miRNAs which can act to reprioritize the patterns of energy production and induce stress-specific cellular adaptations.

One major problem associated with aestivation is oxidative stress and, therefore, for an organism to withstand prolonged periods of aestivation, it must combat oxidative damage. Antioxidant defenses are important strategies used by aerobic organisms to protect macromolecules from damage by reactive oxygen species (ROS). Multiple physiological or environmental stresses can lead to oxidative damage and different combative strategies have been reported in various species that depict the role of antioxidant defenses in protecting animals when challenged by unfavorable environmental conditions (Storey, 1996). One of the problems associated with oxidative stress is lipid peroxidation. High levels of ROS can react with unsaturated bonds in membrane lipids, resulting in lipid peroxidation and causing cellular damage or potentially even leading to death (Catalá, 2009). Disruption of lipid membranes is associated with a plethora of detrimental side effects including disturbance in the fluidity and permeability of the membranes, imbalances in ion and metabolite transportation and interruption of various cellular processes (Borchman et al., 1992; Goldstein & Weissmann, 1977; Kourie, 1988; Mattson et al., 1999). Moreover, lipid peroxidation can negatively affect mitochondrial functions thereby interrupting respiration, membrane potential and calcium buffering abilities (Zhang et al., 1990; Albano et al., 1991).

A recent study has reported that lipid peroxidation is a major contributor to the loss of cellular functions, resulting in oxidative damage during aestivation (Giraud-Billoud et al., 2013). The same study concluded that an effective antioxidant response during the aestivation/recovery cycle was responsible for protecting the midgut of the South American apple snail, *Pomacea canaliculata*. Hence, we proposed that the regulation of key enzymes of fatty acid metabolism during estivation would contribute to the adaptive responses needed to protect under environmental conditions that can cause oxidative stress. EHHADH (enoyl-CoA hydratase and 3-hydroxyacyl CoA dehydrogenase) is a bi-functional enzyme that is a crucial participant in the classical peroxisomal fatty acid *β*-oxidation pathway, the primary pathway involved in lipid catabolism (Houten et al., 2012). There are four enzymatic steps in each cycle of peroxisomal *β*-oxidation: oxidation, hydration, dehydrogenation and thiolytic cleavage (Poirier et al., 2006). EHHADH participates in both the second and third steps of long-chain dicarboxylic fatty acid (DCA) *β*-oxidation (Vamecq & Draye, 1989). Recent evidence suggests that EHHADH is essential for the production of medium-chain DCAs (Houten et al., 2012) that can inhibit mitochondrial respiration by affecting the electron transport chain thereby reducing ATP production while elevating ROS levels (Passi et al., 1984; Tonsgard & Getz, 1985).

Our studies have demonstrated that miRNA-200-3p may play important roles in global transcriptional suppression during aestivation. Preliminary bioinformatics analysis suggested that the *AjEHHADH* transcript could be a target of miRNA-200-3p (Chen et al., 2013). Our present work aimed to elucidate the potential role of miRNA-200-3p in inhibiting oxidative damage during aestivation in the sea cucumber though its actions in regulating *AjEHHADH* gene expression. The present study is the first to report alterations in the transcriptional and translational expression of the intestinal *AjEHHADH* enzyme in the sea cucumber during aestivation. In vivo and *in vitro* analysis of the functional relationship between miR-200-3p and *AjEHHADH* were assessed. Our analysis depicts a preliminary relationship between the involvement of microRNAs in regulating fatty acid metabolism and the reduction or avoidance of lipid peroxidation damage in the sea cucumber during aestivation.

## Materials and Methods

### Animals

Adult sea cucumbers (males and females mixed) (*A. japonicus),* 100 ± 8g, were collected from the coast of Qingdao by diving and hand fishing (Jiaozhou Bay of the Yellow Sea, China). Two groups of sea cucumbers were sampled and dissected right after capture. Non-aestivating sea cucumbers (NA) which served as the control group were sampled in May when the seawater temperature was about 15 °C. At this time and temperature, the sea cucumbers have already recovered fully from aestivation. Animals in deep-aestivation (DA) were sampled in mid-August when seawater temperature was above 25 °C. These animals were sampled after about 15 days of continuous aestivation as indicated by cessation of feeding and locomotion, and the degeneration of the intestine into a very thin and small string (about 2 mm). At each stage (NA and DA), 10 individuals were sacrificed and intestine tissues were dissected, cleared of content and flash frozen in liquid nitrogen. All samples were kept at −80 °C for later analysis. Sea cucumbers (*A. japonicus*) are commercially cultured animals. The study protocol was approved by the Experimental Animal Ethics Committee of the Ocean University of China.

**Table 1:** Primers used in this study.

	Name	Primer Sequences (5′–3′)	Location
RACE	*AjEHHADH*-R1	GCGAGAATGGCGGAGGGTTTGCAAGT	1,382–1,407
	R1-NEST	CAGGTATCCCACTGGCGATGAAACA	1,125–1,149
qRT-PCR	miR-200-3p-F	TAATACTGTCTGGTGATGATG	
	miR-200-3p-R	mRQ 3′ primer	
	5.8s-F	ATCACTCGGCTCGTGCGTC	
	5.8s-R	GCCATTTGCGTTCGAATAAGT	
	*AjEHHADH*-F	GCTCTTTTCTTCTCTGGCCA	954–973
	*AjEHHADH*-R	ACAGGTATCCCACTGCTGAT	1,131–1,150
	*β- Tubulin*-F	GAAAGCCTTACGACGGAACA	
	*β- Tubulin*-R	CACCACGTGGACTCAAAATG	
In vivo	miR-200-3p mimics-F	UAAUACUGUCUGGUGAUGAUG	
	miR-200-3p mimics-R	UCAUCACCAGACAGUAUUAUU	
	Negative control-F	UUCUCCGAACGUGUCACGUTT	
	Negative control-R	ACGUGACACGUUCGGAGAATT	
Dual-luciferase	*AjEHHADH*-WT-F	CGGATCCGACCCTGTAACAACTCTTAACCTCT	2,025–2,048
	*AjEHHADH*-WT-R	CGGATCCGCAACTGACAATTTCTTGTAA	2,280–2,299
	*AjEHHADH*-MUT-F	TTCACCAGGTCATAAAACGAGATTTATTTGATC	2,133–2,165
	*AjEHHADH*-MUT-R	TAGAGCAATTATGACGACCACTTTAAAGAA	2,126–2,155

### RNA extraction, *AjEHHADH* cDNA cloning, and sequence analysis

Total RNA was isolated from intestinal tissues of sea cucumbers using Trizol (Cat No. J20921; TransGen Biotech, Beijing, China) following manufacturer’s instructions. RNA concentration and quality were determined using an Agilent 2100 bioanalyzer. Gene-specific primers for cloning the full-length cDNA of *AjEHHADH* were designed based on the partial sequences of *AjEHHADH* genes obtained from our previously constructed transcriptome library of *A. japonicus* (Data S11). The full-length cDNA sequences were cloned using the SMART RACE cDNA Amplification kit (Cat No. ST0258; Clontech, Mountain View, USA) in accordance with the manufacturer’s instructions. The primer sets for *AjEHHADH* 5′/3′ RACE are listed in Table 1. Polymerase chain reaction (PCR) amplification was carried out using Advantage 2 polymerase mix (Cat No. S1798; Clontech, Mountain View, CA, USA) in a volume of 50 µL. PCR was performed under the following conditions: 95 °C for 1 min; five cycles of 94 °C for 30 s, 66 °C for 30 s, and 72 °C for 3 min; five cycles of 94 °C for 30 s, 64 °C for 30 s, and 72 °C for 3 min; five cycles of 94 °C for 30 s, 62 °C for 30 s, and 72 °C for 3 min; 20 cycles of 94 °C for 30 s, 60 °C for 30 s, and 72 °C for 3 min, followed by a final cycle of 72 °C for 10 min. The expected PCR products were eluted from a 1% agarose gel using a gel extraction kit (Cat No. D2500-02; Omega, Norcross, GA, USA) following the manufacturer’s instructions, then cloned into the pMD19-T vector (Cat No. 6013; Takara, Kusatsu, Japan) and transformed into JM109 competent cells (Cat No. 9057; Takara, Kusatsu, Japan) following the manufacturer’s instructions. Transformed cells were cultured overnight on Luria-Bertani (LB) agar plates containing 100 µg/mL ampicillin. White clones were chosen and cultured in SOC medium (Cat No. HBDC002; Hopebio, Qingdao, China) containing 100 µg/mL ampicillin for 10 h at 37 °C. Positive recombinant clones were sequenced by Sangon Biotech (Shanghai, China). The sequences were analyzed and assembled using DNAStar software (DNAStar Inc., Madison, WI, USA) to identify the open reading frame (ORF). The deduced amino acid sequence of sea cucumber *AjEHHADH* was analyzed using an on-site program http://www.bio-soft.net/sms/index.html. The functional sites or domains in the amino acid sequence were predicted using the simple modular architecture research tool (SMART) version 4.0 (http://smart.embl-heidelberg.de/) and Interpro (http://www.ebi.ac.uk/interpro/).

### Prediction of the miR-200-3p target

Based on our previous transcriptome (Data S11) and proteome (Chen et al., 2016) data for *A. japonicus*, potential miR-200-3p targets were predicted using TargetScan and miRanda software. The target genes for different miRNAs were predicted by the TargetScan algorithm complying with the criteria in the seed region: no mismatch between two to eight nt on the end of miRNA (7mer-m8) The potential targets of miRNA-200-3p were also predicted using the Miranda toolbox based on the complementary region between the miRNA and the 3′-UTR of mRNA, as well as the thermodynamic stability of the miRNA-mRNA duplex. The candidate targets with S >90 (single-residue pair scores) and a minimum free energy lower than −20 kcal/mol were selected for further analysis. The TargetScan program searches for microRNA binding sites (seed matches) conserved between several organisms.

### Dual-luciferase reporter assays

Both wildtype (WT) and mutant (MT) segments of the *AjEHHADH* 3′-UTR (about 500 nt before and after the binding sites) were cloned into pmiR-RB-REPORT luciferase reporter vectors (Ribobio, Guangzhou, China). The primers selected are listed in Table 1. For the transfection experiment, the 293T cells were plated into 96-well white plates 24 h before transfection. Plasmids constructed with pMIR-REPORT vectors were cotransfected with a control Renilla luciferase plasmid (pRL-CMV) (Cat No. E2261; Promega, Madison, WI, USA). The ratio of experimental plasmid to control plasmid was 5:1. Then vectors with 3′ UTR of *AjEHHADH* (WT and MT) were cotransfected with miRNA-200-3p mimics or a negative control. Luciferase assays were performed using the Dual-Luciferase Reporter Assay System (Cat No.E1910; Promega, Madison, WI, USA). In brief, 48 h after transfection, cell lysates were prepared by incubating with 1 × passive lysis buffer for 15 min at room temperature. Cell lysates were transferred to 96-well plates and analyzed using the luciferase dual reporter assay kit (Cat No.E1910; Promega, Madison, WI, USA). The firefly luciferase values were normalized to Renilla.

### *qRT-PCR* analysis of miRNA-200-3p and AjEHHADH, Western blotting analysis of AjEHHADH

Total RNA was isolated as described above from sea cucumber intestinal tissues of the control and estivated groups. RNA concentration and quality were determined using an Agilent 2100 bioanalyzer and then stored at −80 °C for further analysis. The Mir-X miRNA First-Strand Synthesis Kit (Cat No. 638313; Takara, Kusatsu, Japan) was used to synthesize first-strand cDNA as the qRT-PCR template. The entire mature miR-200-3p sequence was synthesized as the specific forward primer (miR-200-3p-F in Table 1) for qRT-PCR and the mRQ 3′ primer supplied with the kit was used as the reverse primer. The 5.8s rRNA was selected as the internal control (see Table 1 for primer sets). SYBR^®^ Premix Ex TaqTM (Cat No. RR820; Takara, Kusatsu, Japan) master mix was used to determine the expression levels of miR-200-3p in both control and estivated groups using the StepOnePlus system (ABI Inc., Foster City, CA, USA). Each sample was run in triplicate along with the internal control gene. Melting-curve analysis of the amplification products was performed at the end of the PCR reaction to determine the purity of the PCR product. The 2^−△△CT^ method was used to analyze the comparative expression levels.

Total RNA was isolated as described above from the intestine of five individuals for both control and estivated groups. First-strand cDNA was synthesized using the M-MLV reverse transcriptase enzyme (Cat No. RR047A; Takara, Kusatsu, Japan) by qRT-PCR. The *AjEHHADH*-specific primer pairs for qRT-PCR were designed based on the obtained open reading frame (ORF) using an online version of Primer 3.0 (Table 1). *β*-tubulin was previously shown to remain unchanged during aestivation in *A. japonicus* and therefore we used it as the internal control (Zhao et al., 2014). Gene expression levels of *AjEHHADH* were analyzed using SYBR^®^ Premix Ex Taq™ master mix (Cat No. RR420; Takara, Kusatsu, Japan) and amplification was measured by the StepOnePlus system (ABI Inc., Foster, CA, USA). Each sample was run in triplicate along with the internal control. Melting-curve analysis of the amplified products was performed at the end of each PCR reaction to ensure that only one PCR product was produced. The 2^−△△CT^ method was used to analyze the comparative expression levels.

Protein extraction and Western blot analysis were performed as previously described (Chen et al., 2016). In brief, total protein was extracted from intestinal tissues using Cell Lysis buffer (Cat No. P0013; Beyotime, Shanghai, China) following the manufacturer’s instructions. The protein concentration was measured using an enhanced BCA protein assay kit (Cat No. P0010S; Beyotime, Shanghai, China) and detected by the SpectraMax Plus system (Molecular Devices, San Jose, CA, USA). The positive control (PC) and the negative control were all set. For 10% SDS-PAGE analysis, 13 µg of protein was loaded and electrophoresed until an appropriate separation was reached. The separated proteins were then transferred to polyvinylidene fluoride membranes (PVDF membrane) (Cat No. IPVH00010; Millipore, Bedford, MA, USA) using wet transfer (BIO-RAD, Singapore). After blocking with 5% non-fat milk in TBS-Tween 20 buffer (TBST) for 2 h at room temperature on a shaker, the membranes were incubated with rabbit polyclonal AjEHHADH antibody (1:600, prepared by Abmart, Shanghai, China) or *β*-tubulin antibody (1:1,000, CST, Cat No. 2146S) overnight at 4 °C. The brief protocol for AjEHHADH antibody development was as follows. Two conserved fragments with antigenic determinant using MacVector version 9 (MacVector Inc., Cambridge, UK) were designed based on the ORF. Constructed plasmids containing the specific sequences were extracted and purified as previously described ( Moore et al., 2015), then injected into two healthy rabbits *in vivo* per antigen. Rabbits were euthanized after seven days and whole blood was withdrawn. Purified antibodies were retrieved using Protein A/G-Plus Beads (Shang Hai Yue-ke Biotechnology CO., Shanghai, China) followed by Elisa verification. The membranes were washed with TBST buffer five times for 5 min each and then incubated with goat anti-rabbit IgG labeled with HRP (1:10,000; Cat No. 7074S; CST, Danvers, MA, USA) for 1 h at room temperature. After washing with TBST for five times (5 min each), the membranes were incubated in ECL Western blot detection reagents (Cat No. P0018; Beyotime, Shanghai, China) and detected using the photo processing machine. Bands were quantified using the Image-Pro Plus 6.0 software (Media Cybernetics Inc., Rockville, MD, USA).

### Functional analysis of miRNA-200-3p *in vivo*

Mimics and negative controls for miR-200-3p were synthesized by GenePharma (Shanghai, China) as shown in Table 1. MiR-200-3p mimics were dissolved in RNase-free water to obtain a working solution of 20 mM. Aliquots of 10 µL of mimics or negative control were mixed with an equal volume of transfection reagent (Beyotime, Shanghai, China) and 80 µL of PBS to serve as the working solution (Lu et al., 2015). Eighteen sea cucumbers (100 ± 8 g) were injected with 100 µl of miR-200-3p mimics or the negative control and a booster injection was given after 24 h. After a further 24 h, treated and control animals were sacrificed and intestine tissues were collected and stored in liquid nitrogen for expression analysis using the qRT-PCR and Western blot protocols described above. The assays were performed with three biological and three technical replicates.

### Statistics

qRT-PCR data are given as mean ± S.E. (*N* = 5) and were analyzed using one-way analysis of variance (ANOVA) followed by a Tukey’s post hoc test (SPSS 17.0 software, Chicago, IL, USA). The level of statistical significance was set at *P* < 0.05 unless otherwise stated. Relative protein abundance was analyzed using a *t*-test (SPSS 17.0) (Chicago, IL, USA) and all the results are given as mean ± S.E. (*N* = 4) .

Dual-luciferase reporter data are given as mean ± S.E. (*N* = 6), qRT-PCR data are given as mean ± S.E. (*N* = 5) and relative protein abundance is given as mean ± S.E. (*N* = 3). These data were used to demonstrate the interaction between miRNA-200-3p and the *AjEHHADH* mRNA transcript. All data were analyzed using an independent sample *t*-test by SPSS 17.0 software. The level of statistical significance was set at *P* <  0.05. *P* < 0.001 indicated an extremely significant difference.

## Results

### Sequence characterization of AjEHHADH and target identification of miR-200-3p

**Figure 1:**
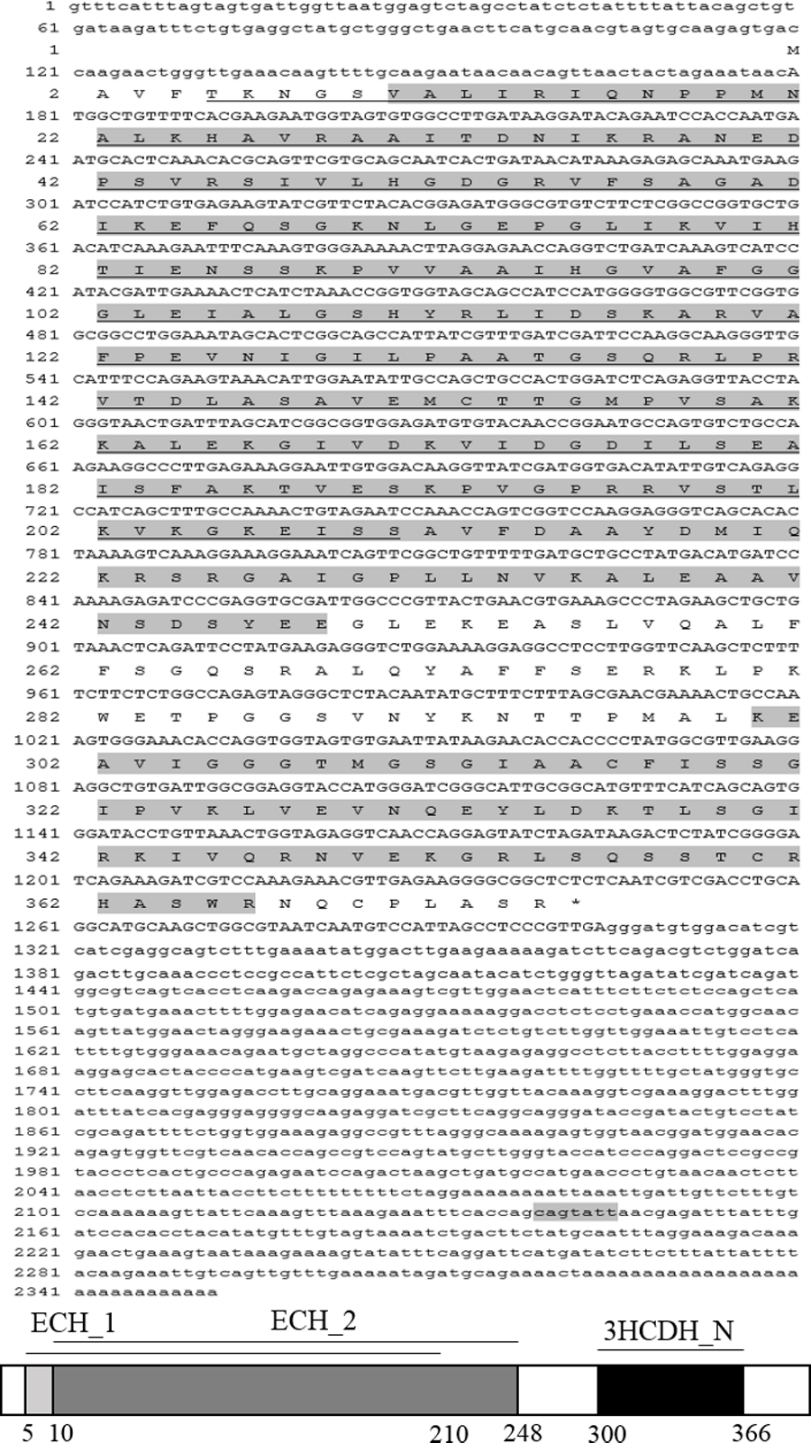
The complete cDNA sequence and deduced amino acid sequence of *AjEHHADH* from *A. japonicus.* Amino acids are numbered starting with the N-terminal Met residue. The asterisk indicates the translational termination codon. The open reading frame (ORF) from the initiation codon (ATG) to the termination codon (TAG) is notated by uppercase letters. At the bottom of the page is the schematic diagram of domains and characteristic motifs.

**Figure 2:**
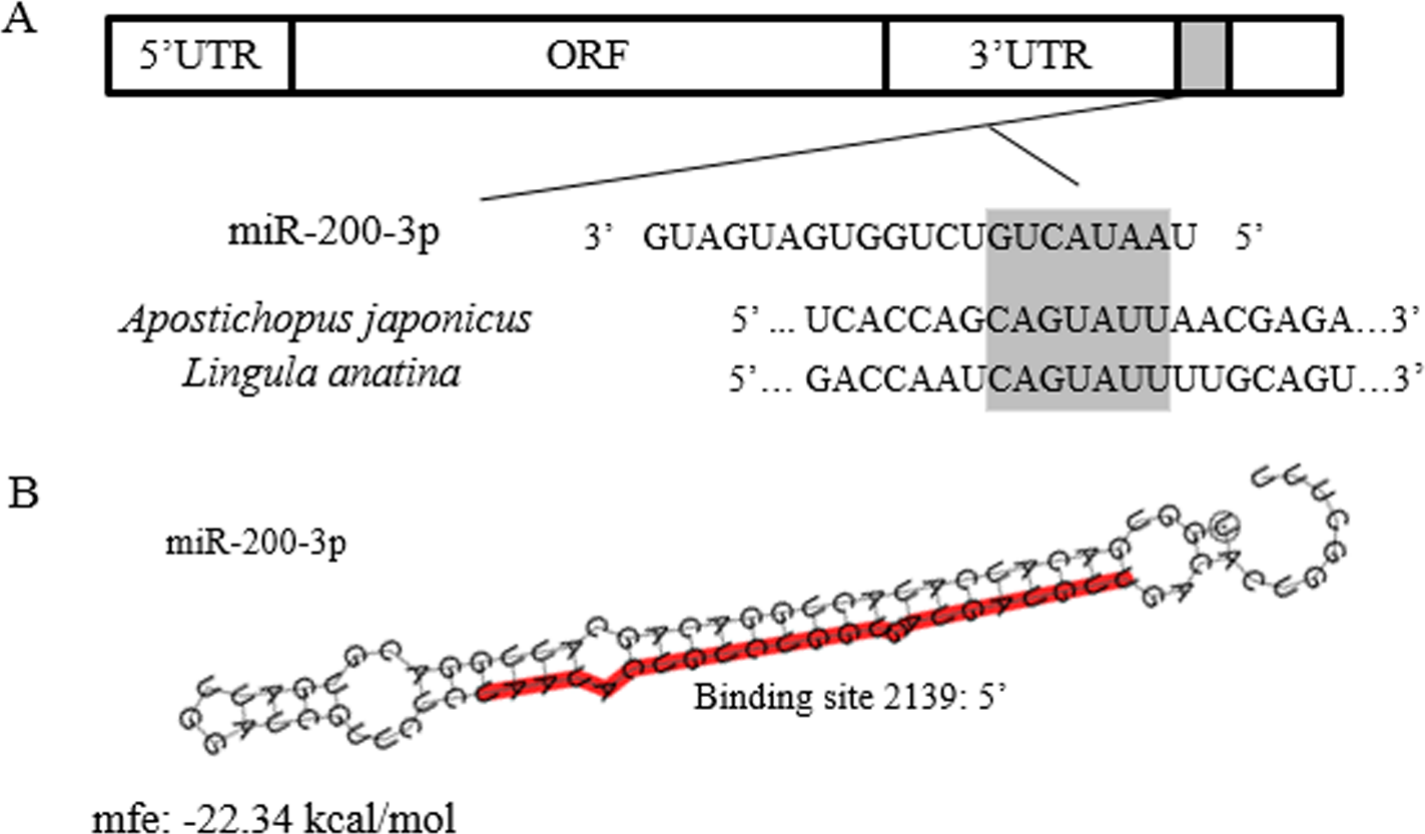
Theoretical binding of miR-200-3p to a conserved region in the 3′ UTR of the *AjEHHADH* gene. (A) Conservation analysis of the miR-200-3p binding site in the *EHHADH* gene from sea cucumber *A. japonicus* and *Lingula anatina*. The seed region sequence (shaded) shows 100% conservation between these two sequences. (B) Predicted binding structure of miR-200-3p when binding to the 3′ UTR of *AjEHHADH,* as determined from the TargetScan and miRanda program.

The full-length cDNA of *AjEHHADH* spanned 2,352 bp, including a 179 bp 5′-UTR and a 1,048 bp 3′ UTR. The open reading frame (ORF) contained 1,125 bp and encoded a 375 long amino acid polypeptide with a calculated molecular weight of 67 kDa (Fig. 1). SMART analysis indicated that the sea cucumber *AjEHHADH* protein contained two enoyl-CoA hydratase/isomerase (ECH) domains and a 3-hydroxyacyl-CoA dehydrogenase NAD binding domain (Fig. 1) (Gene accession No. KY328747KY). Analysis of the putative miR-200-3p binding sites was then performed using TargetScan 5.2 and Miranda 3.3a programs. Results showed that the 3′ UTR of sea cucumber *AjEHHADH* contained binding sequences for miR-200-3p starting from residue 2,139 within the 3′ UTR sequence. Importantly, the binding site was also identified and found to be conserved in *Lingula anatine*, a member of another marine invertebrate group, the brachiopods (Fig. 2). The theoretical minimum free energy of binding (kcal/mol) was also calculated using the program Miranda. Theoretical prediction of thermodynamic binding parameters for miRNA-200-3p binding to *AjEHHADH* was calculated to be −22.34 kcal/mol (below the given theoretical parameters −20 kcal/mol), suggesting that *AjEHHADH* is a likely target of miRNA-200-3P in sea cucumbers (Fig. 2).

### The expression of miR-200-3p and AjEHHADH during aestivation

**Figure 3:**
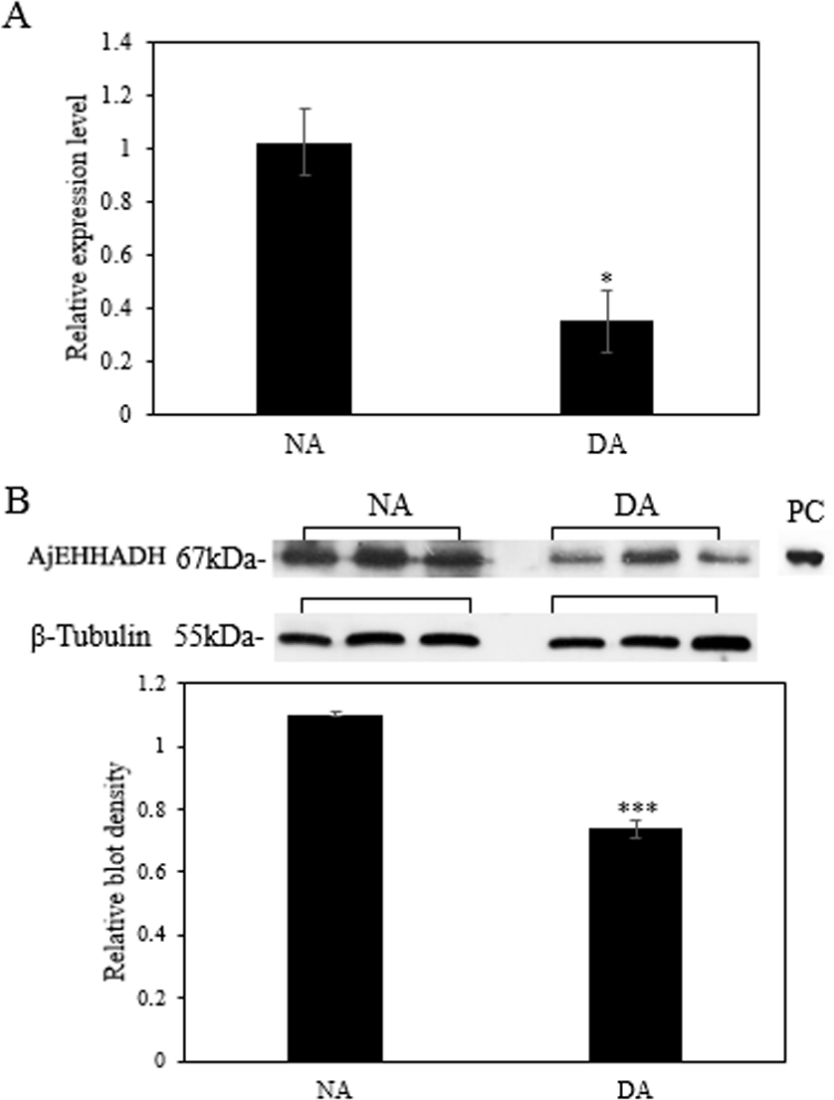
Effect of estivation on the relative expression levels of *AjEHHADH*. (A) Relative mRNA expression levels of *AjEHHADH* in the intestine of NA and DA groups respectively. Values were normalized against *β*-Tubulin. “*” indicates significant statistical differences (*P* < 0.05). Values are means ± SE (*N* = 5). (B) Relative protein expression level of AjEHHADH at the NA and DA stages in intestine by Western blot. Representative bands show blot intensity for NA and DA groups. Histograms show normalized expression levels for NA and DA. *β*-Tubulin was chosen as the internal control. “***” indicates significant differences for NA and DA groups (*P* < 0.001). Values are means ± SE (*N* = 4).

**Figure 4:**
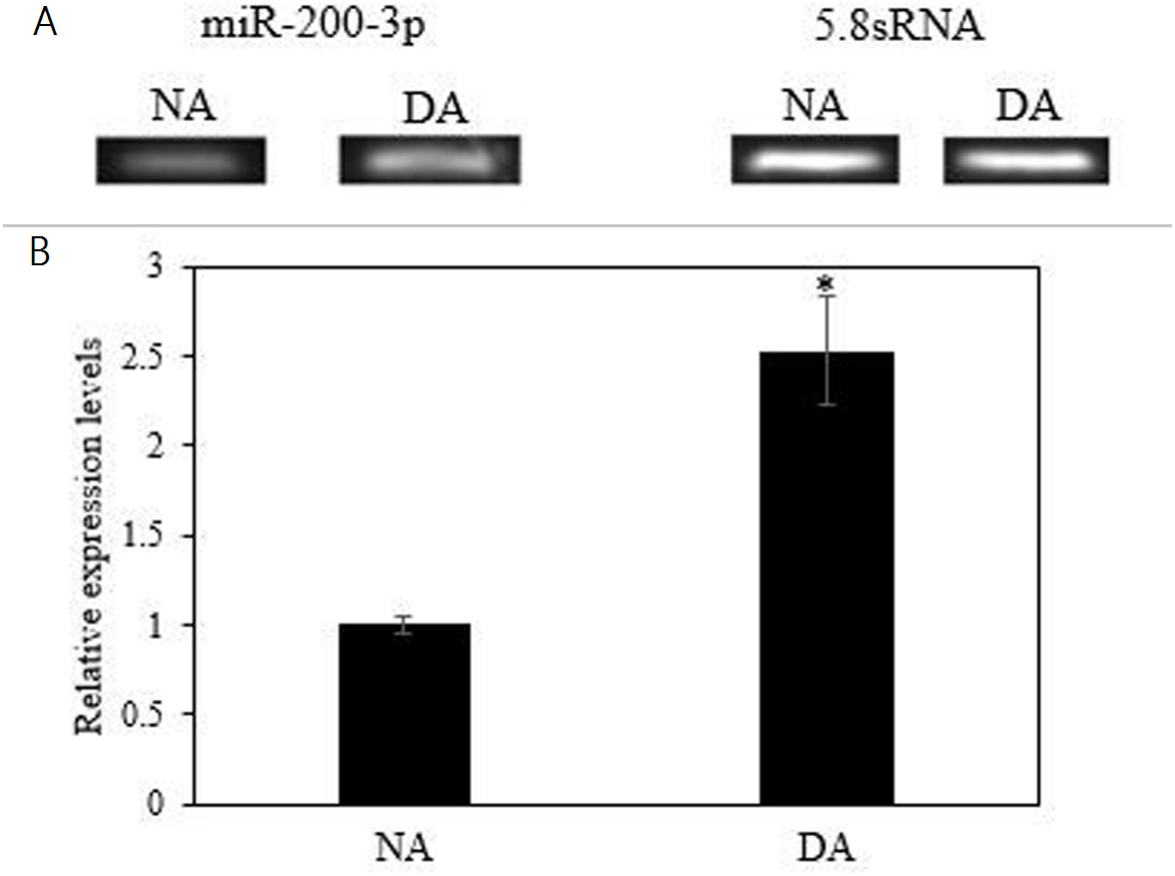
Effect of estivation on the relative expression of miRNA-200-3p. Representative bands show RNA transcript levels amplified by RT-PCR. Band intensities from the RT-PCR samples were normalized to either 5.8S rRNA (microRNA) band amplified from the same sample. Data are means ± SE (*N* = 5 independent trials on tissue from different animals). “*” Indicates a significant difference from the corresponding control (*p* < 0.05).

The mRNA transcript levels of *AjEHHADH* decreased significantly in the intestine by 0.34 ± 0.11-fold at the DA stage compared to the NA stage (*P* < 0.05) (Fig. 3A). Moreover, the protein expression levels of *AjEHHADH* also showed a significant decrease by 0.72 ± 0.08-fold at the DA compared with NA stage (*P* < 0.05) (Fig. 3B). The expression of miR-200-3p was assessed in the intestine of *A. japonicus* from non-aestivation and deep-aestivation stages. MiR-200-3p expression levels showed an inverse correlation with *AjEHHADH* transcript and protein levels in the intestine. Expression levels of miR-200-3p increased significantly in the intestine by 2.53 ± 0.3-fold in the deep-estivated group compared to controls (*P* < 0.05) (Fig. 4).

### Validation of the interaction between 3′ UTR of AjEHHADH and miR-200-3p by dual-luciferase reporter assays

**Figure 5:**
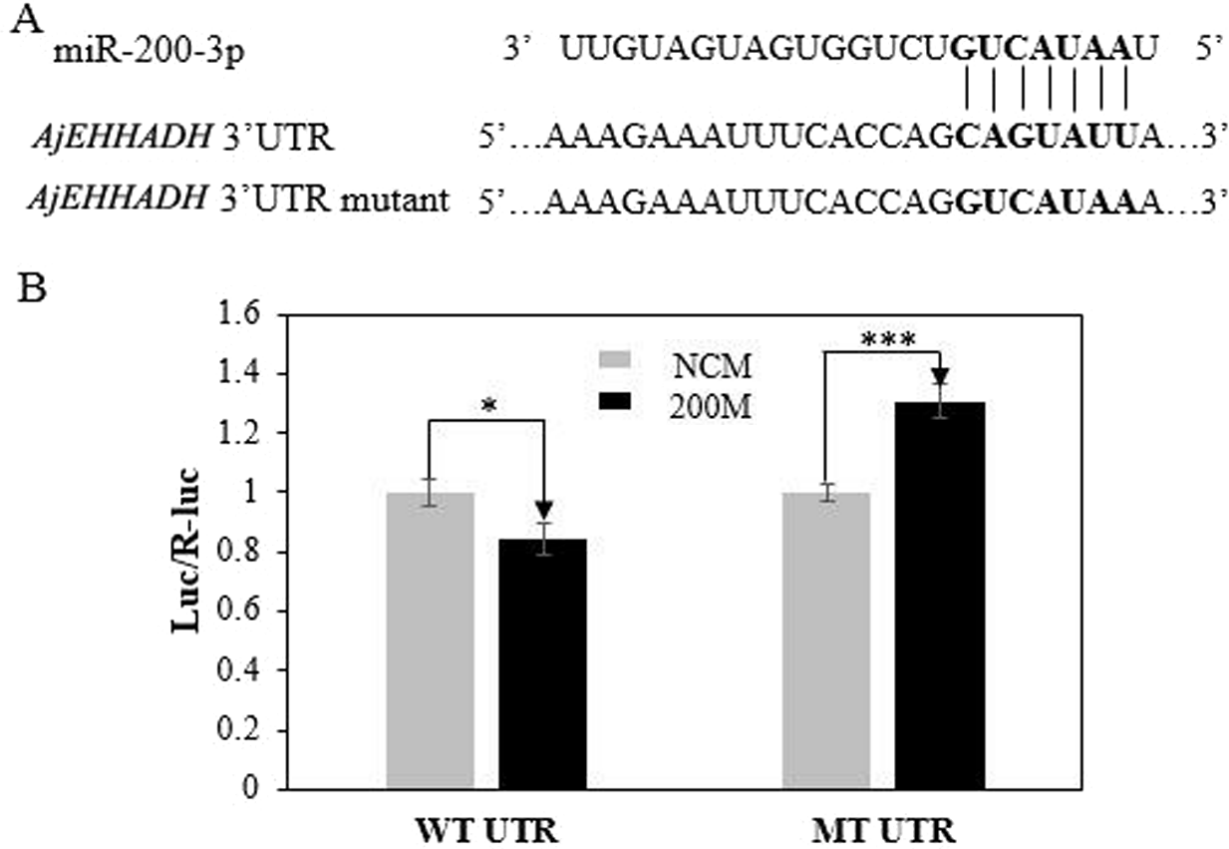
Identification and characterization of the miRNA-200-3p binding sites in the 3′ UTR of *AjEHHADH* and functional effect of miR-200-3p on *AjEHHADH*. (A) Schematic representation of the putative miRNA-200-3p targeting sites in *AjEHHADH* mRNA and the respective mutant sites. (B) HEK-293T cells were co-transfected with the pMIRREPORT-BHMT-WT vector, carrying the wild-type and the mutated *AjEHHADH* 3′-UTR, pRLCMV-Renilla-luciferase, and control miR-200-3p mimics as indicated. “*”indicates significant differences (*P* < 0.05). WT, Wild-type; MT, Mutant type; 200M, miR-200-3p mimics; NCM, negative control without miR-200-3p.

To further validate the predicted role of miR-200-3P in regulating *AjEHHADH*, plasmids containing the putative binding site for miRNA-200-3p and the mutation site (Fig. 2) of the 3′ UTR of *AjEHHADH* were constructed and used in the classical reporter assay. Information concerning the binding and mutation sites of miRNA-200-3p in the 3′ UTR of *AjEHHADH* is shown in Fig. 5A. A significant reduction (11.9%, *P* = 0.044) was observed in luciferase activity transfected with WT UTR compared with control (Fig. 5B).

### MiR-200-3p modulates AjEHHADH at the post-transcriptional level *in vivo*

**Figure 6:**
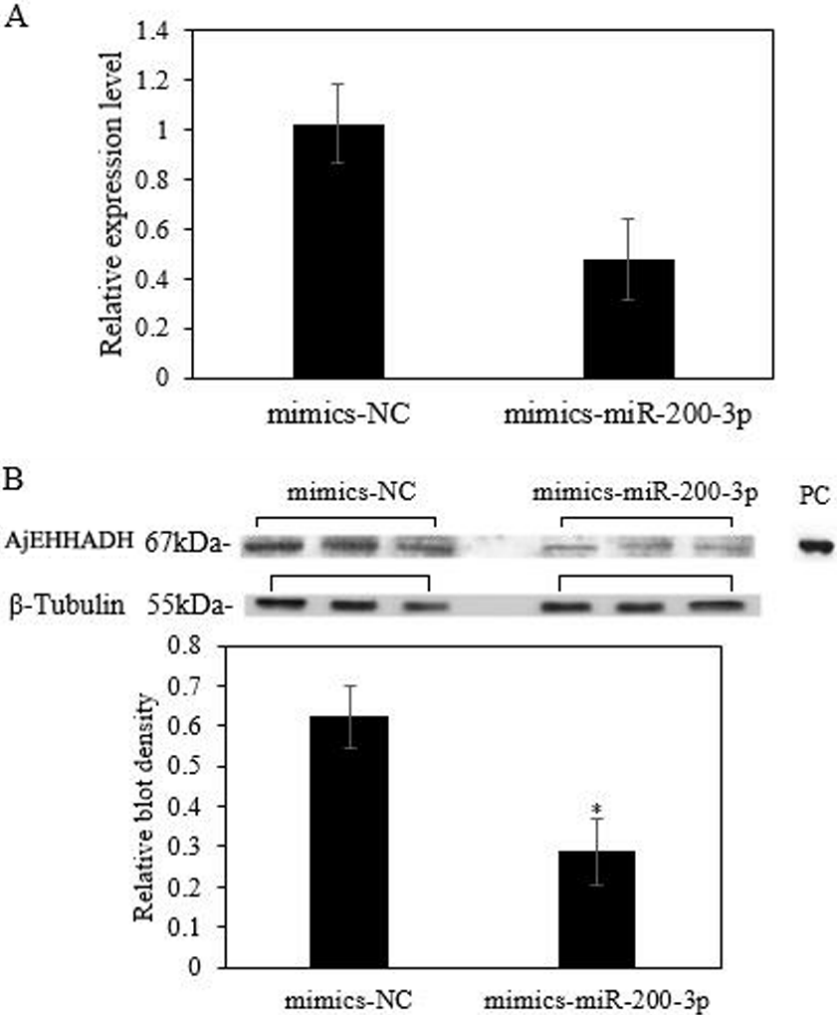
Functional analysis of miR-200-3p and *AjEHHADH in vivo*. (A) Relative *AjEHHADH* mRNA transcripts expression after transfection with miRNA modified mimics. (B) Western blot analysis after transfection with miRNA modified mimics. “*” indicates significant differences (*P* < 0.05). Values are means ± SE (*N* = 3 for mRNA and protein analysis).

To fully understand the potential roles of miR-200-3p in regulating *AjEHHADH*, gain-of-function assays for miR-200-3p were performed *in vivo*. As shown in Fig. 6A, the qRT-PCR results indicated that overexpression of miR-200-3p decreased the transcript levels of *AjEHHADH*, although it was not significant in statistical analysis (*P* > 0.05). Western blot analysis revealed that AjEHHADH protein levels were also reduced significantly upon miR-200-3p overexpression (Fig. 6B), consistent with the *AjEHHADH* transcript expression levels (Fig. 3). These results indicate that miR-200-3p may directly target *AjEHHADH* gene expression by inducing the degradation of the *AjEHHADH* mRNA transcripts.

## Discussion

Recent studies have shown that miRNA-mediated gene silencing is a widespread regulatory mechanism influencing many processes during periods of environmental stress including the cell cycle, apoptosis, signal transduction, muscle atrophy and fatty acid metabolism (Kornfeld, Biggar & Storey, 2012; Wu, Biggar & Storey, 2014; Biggar, Dubuc & Storey, 2009; Biggar & Storey, 2012). Accumulated evidence shows that miRNAs play a significant role in metabolic rate depression where they can modulate the availability of corresponding transcripts, thereby providing a method for maintaining energy expenditure in check.

To date, very little is known about the potential stress-responsive roles of miRNAs in targeting specific genes in non-model marine invertebrates like the estivating sea cucumber. Using RNA-seq and microRNA microarrays, we have previously shown the involvement of miR-200-3P in the intestine of the sea cucumber whereby expression levels increased significantly during deep aestivation compared to the non-estivated control group (Chen et al., 2013). To further understand the regulatory influence of miR-200-3p in the estivating sea cucumber, potential target genes were predicted. Our prediction showed that the *AjEHHADH* gene, corresponding to a protein involved in lipid peroxidation could be targeted by miR-200-3p; therefore, we aimed to investigate this prediction further.

MicroRNAs are known to regulate translation by regulating the availability of mRNA to the translational machinery, thereby providing a finely tuned mechanism for regulating protein translation. The present study identified *AjEHHADH* as a novel target of miR-200-3p using bioinformatics analysis. To confirm our predictions, we explored the expression patterns of *AjEHHADH* gene and protein during aestivation using qRT-PCR and Western blot in the sea cucumber. The results indicated a negative relationship between miR-200-3p and *AjEHHADH* expression at both the transcriptional and the translational levels, suggesting that *AjEHHADH* could be regulated by miR-200-3p. Dual-luciferase reporter assays further identified the interaction between miRNA-200-3p and *AjEHHADH*. To further examine our claims, we performed *in vivo* gain-of-function assays to overexpress miR-200-3p and again explored the gene and protein levels of sea cucumber *AjEHHADH* by qRT-PCR and Western blot, respectively. The results support the claim that miR-200-3p is responsible for regulating the degradation of *AjEHHADH* mRNA transcripts, thereby causing a sharp decline in the abundance of the corresponding protein. This finding further supports our claim and shows that miR-200-3p does indeed play a significant role in inhibiting the expression of *AjEHHADH*.

The EHHADH enzyme has been previously identified as a key player in the classical peroxisomal *β*-oxidation pathway, and is crucial in the formation of the medium-chain DCAs, adipic and suberic acid, as well as their carnitine esters during fasting (Houten et al., 2012). Earlier studies showed that medium-chain DCAs can inhibit mitochondrial respiration thus favoring ROS production (Passi et al., 1984; Tonsgard & Getz, 1985) which can be further exacerbated by peroxisomal *β*-oxidation (Curzio, Esterbauer & Dianzani, 1985). These two findings that are consistent with our observations in this study. Intestinal degeneration and cessation of feeding for prolonged periods of time are well-known characteristics of in sea cucumber aestivation (Li et al., 1996). Such events generally lead to oxidative stress which can damage macromolecules and cause the accumulation of lipid peroxidation products, leading to cellular stress and inflammation (Bikman & Summers, 2011). Here, we propose that miRNA-200-3p may act as a regulatory mechanism to protect cells from oxidative damage by inhibiting the translation of the *AjEHHADH* transcripts when sea cucumbers enter an aestivation-induced hypometabolic state.

In summary, the present study provides the first demonstration of *EHHADH* regulation during aestivation in sea cucumbers. Our results suggest that aestivation-responsive suppression of *AjEHHADH* mRNA and protein levels in the intestine may be subjected to regulation at the post-transcriptional level by miRNA-200-3p. This also indicates *β* that miRNA-200-3p could play a role in regulating fatty acid metabolism, thereby offering cytoprotection to cells and organelles during aestivation in sea cucumbers. Further studies are required to confirm the roles of miRNAs in other aspects of fatty acid metabolism in animals that undergo hypometabolism to deal with environmental stress.

##  Supplemental Information

10.7717/peerj.5703/supp-1 Supplemental Information 1 Part of the sequencing chromatogram of *AjEHHADH* gene Representative sequencing chromatogram of *AjEHHADH* gene, different colors of evenly-spaced peak represent different nucleotide bases.

10.7717/peerj.5703/supp-2 Supplemental Information 2 Another part of the sequencing chromatogram of *AjEHHADH* gene Representative sequencing chromatogram of *AjEHHADH* gene, different colors of evenly-spaced peak represent different nucleotide bases.

10.7717/peerj.5703/supp-3 Supplemental Information 3 ** The raw data of qRT-PCR result of *AjEHHADH.* ** Raw data of mRNA expression levels of *AjEHHADH* and *β*-Tubulin in the intestine of NA and DA groups respectively.

10.7717/peerj.5703/supp-4 Supplemental Information 4 The western blotting result of *AjEHHADH .* The gel photo of protein expression level of *AjEHHADH* and *β*-Tubulin at the NA and DA stages in intestine.

10.7717/peerj.5703/supp-5 Supplemental Information 5 The raw data of qRT-PCR result of miR-200-3p Raw data of RNA transcript levels of miRNA-200-3p in the intestine of NA and DA groups respectively.

10.7717/peerj.5703/supp-6 Supplemental Information 6 The nucleic acid abundance of 5.8 sRNA and miR-200-3p 1% agarose gel photos of 5.8 sRNA and miR-200-3p.

10.7717/peerj.5703/supp-7 Supplemental Information 7 Raw data of the Dual-luciferase reporter assays Original fluorescence values of Luc and R-Luc in the WT and MT.

10.7717/peerj.5703/supp-8 Supplemental Information 8 The raw data of qRT-PCR result of *AjEHHADH* after transfection with miRNA modified mimics Raw data of relative mRNA expression level of *AjEHHADH* and *β*-Tubulin in the intestine of miR-200-3p mimics groups.

10.7717/peerj.5703/supp-9 Supplemental Information 9 The western blotting result of *AjEHHADH* after transfection with miRNA modified mimics. ** Gel photo of western blot of *AjEHHADH* after transfection with miRNA modified mimics.

10.7717/peerj.5703/supp-10 Supplemental Information 10 The western blotting result of *β*-Tubulin after transfection with miRNA modified mimics. ** Gel photo of western blot of *β*-Tubulin after transfection with miRNA modified mimics.

10.7717/peerj.5703/supp-11 Supplemental Information 11 The intestine transcriptome library of *A. japonicus*

## Additional Information and Declarations

### DNA Deposition

The following information was supplied regarding the deposition of DNA sequences: The sequence described is accessible via GenBank accession number KY328747.

### Data Availability

The following information was supplied regarding data availability: The raw data are provided in the Supplemental Files.
