# Supplementary figures and images for: The potential contribution of miRNA-200-3p to the fatty acid metabolism by regulating AjEHHADH during aestivation in sea cucumber
Source: PeerJ. 2018 Oct 2;6:e5703. doi: 10.7717/peerj.5703 (PMC6173160; doi:10.7717/peerj.5703)

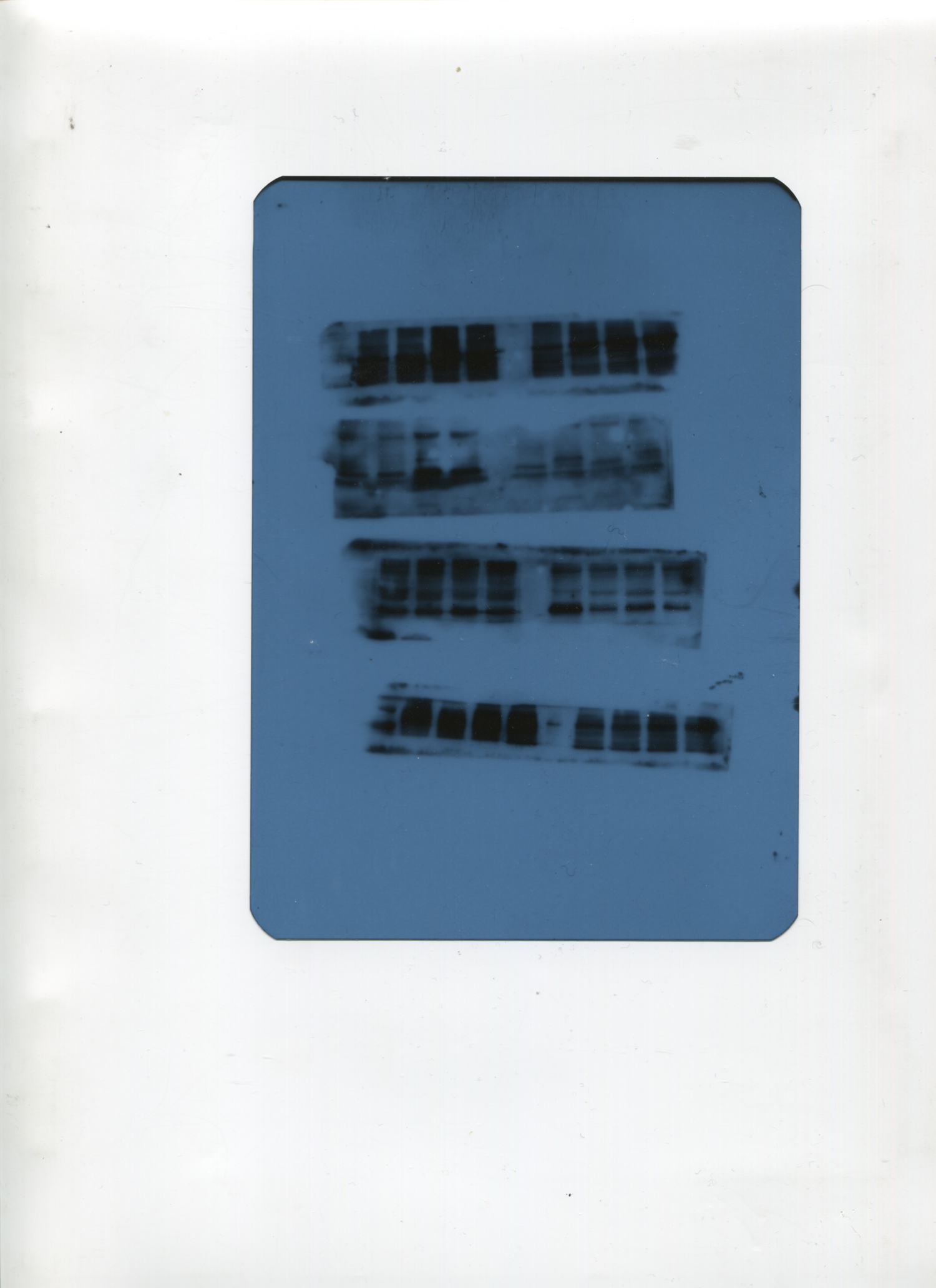

Supplement: Supplemental Information 4 — The gel photo of protein expression level of AjEHHADH and β-Tubulin at the NA and DA stages in intestine. [file peerj-06-5703-s004.png]

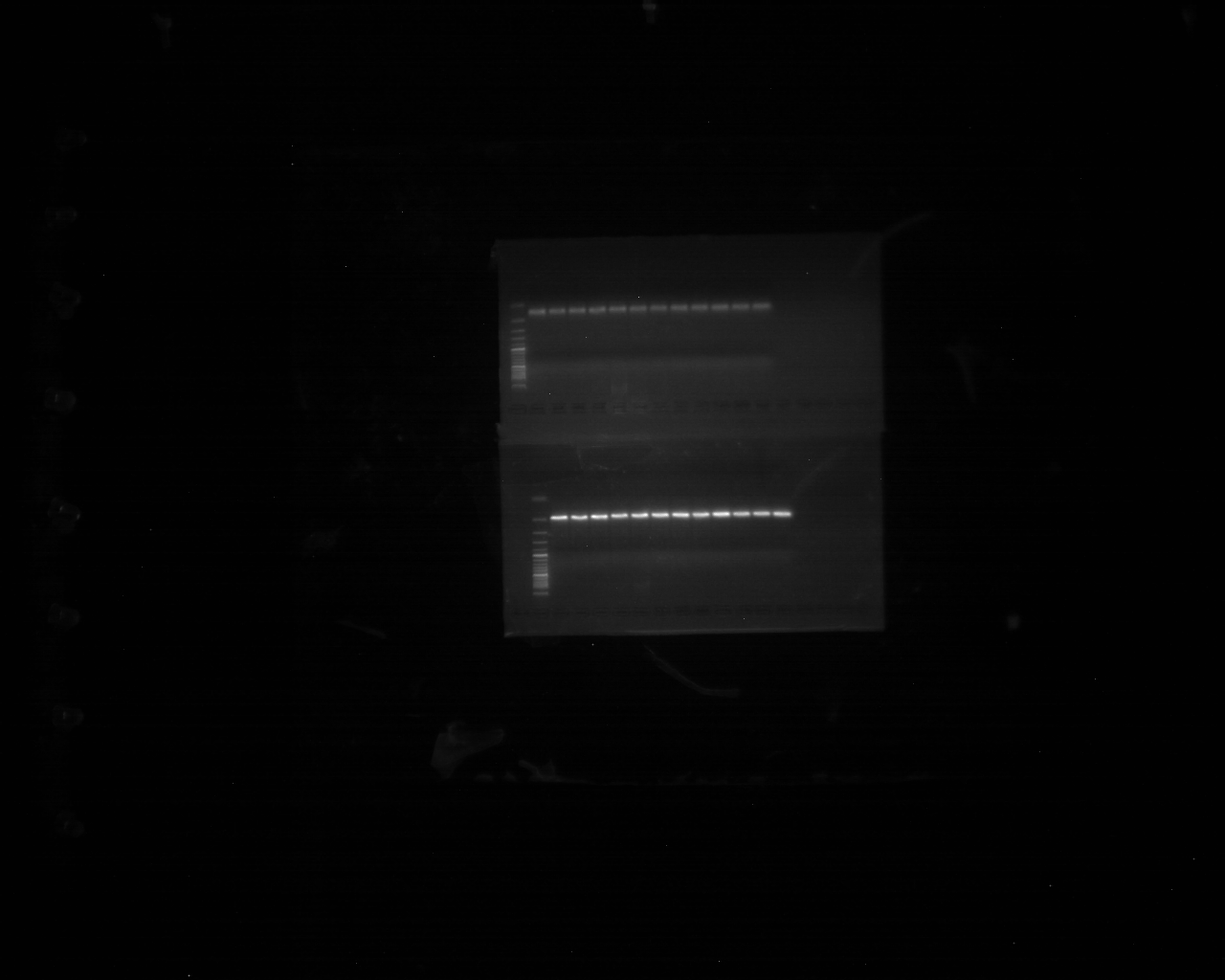

Supplement: Supplemental Information 6 — 1% agarose gel photos of 5.8 sRNA and miR-200-3p. [file peerj-06-5703-s006.png]

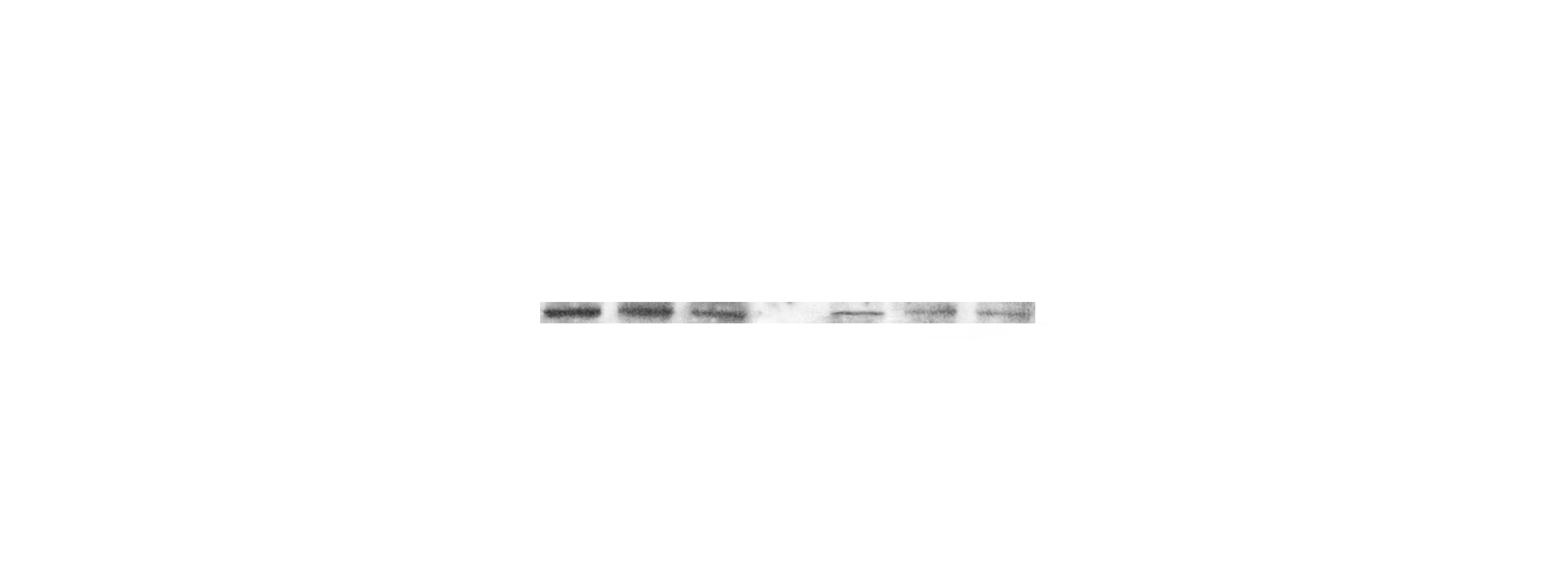

Supplement: Supplemental Information 9 — Gel photo of western blot of AjEHHADH after transfection with miRNA modified mimics. [file peerj-06-5703-s009.png]

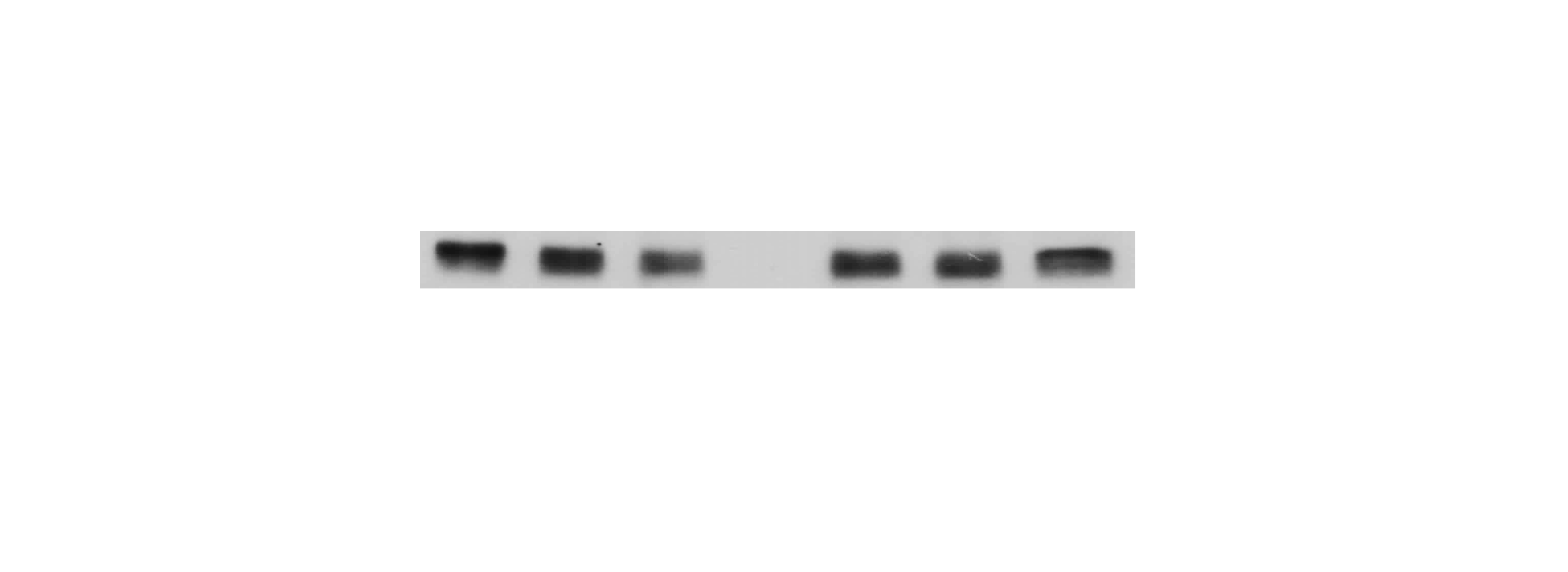

Supplement: Supplemental Information 10 — Gel photo of western blot of β-Tubulin after transfection with miRNA modified mimics. [file peerj-06-5703-s010.png]
